# Risk Factors Contributing to Highly Pathogenic Avian Influenza H5N6 in China, 2014–2021: Based on a MaxEnt Model

**DOI:** 10.1155/2023/6449392

**Published:** 2023-09-15

**Authors:** Zhuo Sun, Yue-peng Li, Qi An, Xiang Gao, Hong-bin Wang

**Affiliations:** ^1^College of Veterinary Medicine, Northeast Agricultural University, Harbin, China; ^2^Key Laboratory of the Provincial Education Department of Heilongjiang for Common Animal Disease Prevention and Treatment, College of Veterinary Medicine, Northeast Agricultural University, Harbin, China

## Abstract

Highly pathogenic avian influenza H5N6, an acute infectious poultry disease, has appeared in China since 2014. The serious HPAI H5N6 epidemic has caused substantial economic losses to the poultry industry and seriously threatened public health. This study developed two maximum entropy models based on highly pathogenic avian influenza virus H5N6 surveillance data obtained through China's national surveillance program and HPAI H5N6 outbreak data in China, identified risk factors for the disease and developed risk prediction maps. Both models had a good predictive performance with area under the receiver operating characteristic curves of 0.931 and 0.892. Our study considered 26 variables from bioclimate, geographical, and socioeconomic factors. After the screening, 14 variables were retained and incorporated into the construction of the two models. HPAIV H5N6 presence was related to population density, road density, poultry market density, mean diurnal range, and chicken density. HPAI H5N6 poultry outbreaks were associated with population density, road density, mean diurnal range, precipitation of the driest month, and chicken density. Overall, Southern China was the high-risk area for HPAIV H5N6 maintenance and H5N6 outbreak. The risk factors and maps associated with the HPAIV H5N6 presence and HPAI H5N6 outbreak identified in our study will help develop appropriate HPAIV H5N6 control measures to reduce the risk of infection and morbidity in poultry and humans.

## 1. Introduction

highly pathogenic avian influenza (HPAI) H5N6 initially emerged in Sainyabuli Province, Laos, on March 13, 2014 [1]. For a long time afterward, highly pathogenic avian influenza virus (HPAIV) H5N6 spread across national borders by migratory birds, causing large outbreaks in Asia [2]. As of September 2022, HPAI H5N6 has affected eight countries in Asia, including China, Vietnam, Laos, Myanmar, Cambodia, Philippines, Korea, and Japan, leading to the deaths of more than 200 thousand poultry and 669 wild birds. Not only that, HPAI H5N6 was introduced to many European countries in 2017 [3, 4], such as the Netherlands, Switzerland, Greece, and the U.K. HPAI H5N6 has the potential to be the next influenza pandemic over the world and a new threat to world health, making it worthy of our attention.

On April 23, 2014, HPAI H5N6 was detected in chickens on a farm in Nanbu County, Nanchong City, Sichuan Province, China, marking the first reported outbreak of HPAI H5N6 in China [5]. As the outbreak unfolded, over 40 outbreaks in poultry and 18 outbreaks in wild birds have affected 18 provinces of China, causing the culling of more than 650 thousand poultry to curb the spread of the disease. Even worse, HPAIV H5N6 appears to have gained the ability of transspecies transmission to humans and induce ongoing human infections and deaths in China [6–8]. It may be attributed to the Chinese preference to purchase live poultry [9], significantly increasing human exposure to HPAIV H5N6.

China has launched a large-scale nationwide vaccination campaign to control HPAI H5N6 and has also intensified postvaccination surveillance, including antibody seroprevalence and virus positivity rate. Simultaneously, the Ministry of Agriculture and Rural Affairs (MOA) in China has been coordinating a national surveillance project to detect the HPAIV H5N6 by routine virological surveillance from samples of chicken, duck, goose, wild birds, swine, and related environments [10]. HPAIV H5N6 detected regularly through the national surveillance program provided evidence that China created a powerful venue for the dynamic recombination of HPAIV H5N6 with other HPAI viruses and the further evolution and spread of HPAIV H5N6 [11]. China has excellent geographical conditions, abundant water resources, a warm subtropical climate, and large-scale, high-density, multimodal poultry farming. Particularly, southern China has traditionally been known as an epicenter for HPAIV H5N6 [12]. However, very few studies have mapped the potential distribution of HPAIV H5N6 and analyzed risk factors favoring the infection and transmission of HPAIV H5N6 and the HPAI H5N6 epidemic in China. Without a suitable description of the distribution characteristics of HPAI H5N6 and the interrelationship between HPAI H5N6 and environmental and socioeconomic indicators, it is difficult to refine the control strategies dependent on the surveillance project and vaccination. Determination and a better understanding of the risk factors associated with HPAI H5N6 and the risk prediction map are critical to epidemic control. Specifically, it would contribute to developing suitable HPAIV H5N6 control measures and strengthen monitoring for the HPAIV H5N6 in high-infection-risk areas of China, thus optimizing resources allocated to controlling the disease and reducing the risk for poultry and human infection.

Previous studies have identified environmental factors associated with HPAI H5N1 and HPAI H7N9, such as wild bird migration [13], lakes and wetlands [10], climate [13], altitude [14], live poultry trade [15], distance to the nearest road [10], and human population density [10, 14]. However, only a few risk factors definitively associated with HPAI H5N6 have been confirmed. The main pathway identified for the spread of HPAIV H5N6 was the migration of wild birds [2, 4]. The live poultry trade has also played an essential role in spreading and maintaining HPAIV H5N6 in China [16]. Furthermore, poultry density was an important determinant of HPAIV H5N6 infection in Korean farm flocks [17]. The respective roles played by other factors in the presence and spread of HPAIV H5N6 and the HPAI H5N6 outbreak in China are still unclear.

Therefore, we selected 26 variables for our study from three aspects: bioclimate, geographical, and socioeconomic factors, and aimed to analyze the different roles played by these factors in the spread and outbreak of HPAI H5N6 in China by building two maximum entropy models. Specifically, it would explore the association of selected variables with two datasets associated with HPAI H5N6: HPAIV H5N6 infections detected through the national surveillance project and reported HPAI H5N6 outbreaks.

## 2. Materials and Methods

### 2.1. Data on HPAI H5N6

Two types of data related to the presence of HPAI H5N6 were collected as dependent variables in this study.

First, samples of chickens, ducks, geese, wild birds, pigs, and associated environments collected at the provincial level through a national surveillance project coordinated by MOA were tested by polymerase chain reaction. All AIV-positive samples were sent to the National Veterinary Research Institute in Harbin for confirmation, subtype identification, and virus isolation. Positive results for HPAIV H5N6 were subsequently reported at the central government level. Data were recorded in the monthly official veterinary bulletin published on the website of the MOA, from which we extracted 253 HPAIV H5N6 positive points from 2014 to 2017. To reduce spatial clustering, the rangeBuilder package in R software was used to filter out the positive points with a filtering range of 1 km, and duplicate points were eliminated. A total of 160 points were included in the final model after data filtering.

Second, data about HPAI H5N6 outbreaks in poultry were derived from two sources: (1) one being the website of MOA (http://www.agri.gov.cn) and (2) the other obtaining from the World Animal Health Information System of the World Organization for Animal Health (https://wahis.woah.org/). Two different sources of poultry outbreaks data were compared and combined into one complete database. A total of 46 points of HPAI H5N6 poultry outbreaks were collected in China from 2014 to 2021. The study was performed under the resolution of 30 arc seconds (approximately 1 km × 1 km), in which only one record point can be kept in a grid cell. Finally, 45 points of HPAI H5N6 were included in the subsequent maximum entropy model construction after data cleaning within the grid cells.

### 2.2. Variables

Twenty-six variables involving bioclimate, geographical, and socioeconomic factors were obtained to establish the models (Table 1).

Nineteen bioclimate variables were downloaded from WorldClim (https://worldclim.org/data/), exhibiting a high resolution of 30 arc seconds (approximately 1 km × 1 km). Elevation and waterbody were considered in this model as geographical factors. Elevation with a high resolution of 30 arc seconds (approximately 1 km × 1 km) was also accessed from WorldClim. Waterbody was obtained from the OpenStreetMap website (https://www.openstreetmap.org/). Five socioeconomic variables were considered: population density, chicken density, duck density, road, and poultry market. Population density was sourced from https://landscan.ornl.gov/. Chicken and duck density were obtained from the Gridded Livestock of the World (http://www.fao.org/livestock-systems/en/). Population density, chicken density, and duck density had a high resolution of 30 arc seconds (approximately 1 km × 1 km). Road was taken from the OpenStreetMap website. Considering poultry transport's impact on HPAI H5N6, only the first three levels of major roads in China were extracted. Data on the distribution of poultry markets in China were crawled from Google Maps via Python. A total of 699 Chinese poultry markets with clear names were obtained after filtering the crawl results.

Waterbody, road, and poultry market collected in shp format were converted into raster format by the kernel density analysis and resampled to 30 arc seconds (approximately 1 km × 1 km). Furthermore, all variables were cropped to the geographical area of mainland China. All operations were accomplished in ArcGIS 10.2.

The variance inflation factors (VIFs) of these variables were calculated to perform a multicollinearity test, using the usdm package in R software [18]. Variables with VIF > 10 were excluded from the MaxEnt model, as their collinearity problem would lead to overfitting of the model [19]. A MaxEnt model for HPAIV H5N6 surveillance positives and a MaxEnt model for HPAI H5N6 poultry outbreaks were developed separately. The variables incorporated in the two models are shown in Table 1.

### 2.3. Establishing the MaxEnt Models

The MaxEnt model is one of the most popular ecological niche models [20], allowing accurate prediction when a small number of species distribution coordinates are available [21]. Two indicators of HPAIV H5N6 were modeled using MaxEnt 3.4.4, with 10 replications of cross-validation [22]. Meanwhile, defined as “pseudo-vanishing” data, 10,000 background points with the same bias were put into two models to reduce sampling bias, a common problem in species distribution models [23].

### 2.4. Model Evaluation and Interpretation

The receiver operating characteristic curve was used to verify the performance of two models [24]. The area under the receiver operating characteristic curve (AUC) ranges from 0 to 1, with larger values showing better predictive performance [25]. Jackknife test of the MaxEnt model also gives the AUC when modeling each variable individually, which is used to assess the importance of each variable [26]. Response curves generated by MaxEnt model reflect the dependence of predicted accuracy on each variable [27]. The risk value of risk prediction maps based on HPAIV H5N6 surveillance positives and HPAI H5N6 poultry outbreaks consisted of values ranging from 0 to 1, representing low to high risk of HPAIV H5N6 presence and HPAI H5N6 outbreak.

## 3. Results

**Figure 1:**
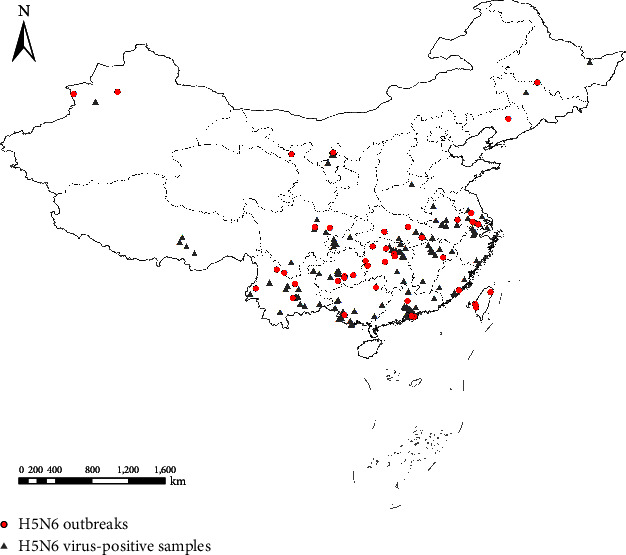
Distribution of HPAI H5N6 virus-positive samples (gray triangles) identified through national surveillance project and HPAI H5N6 outbreaks (red dots) in China.

**Figure 2:**
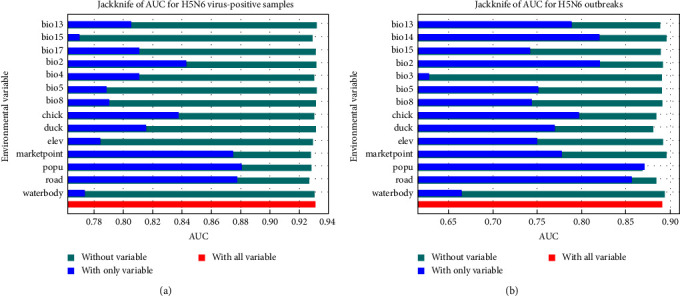
Jackknife test results of AUC for H5N6 virus-positive samples (a) and H5N6 outbreaks (b).

The distribution of HPAIV H5N6 surveillance positives and HPAI H5N6 poultry outbreaks are shown in Figure 1. MaxEnt models built in this study performed well with AUCs of 0.931 and 0.892 for HPAIV H5N6 surveillance positives and HPAI H5N6 poultry outbreaks, respectively. The accuracy was higher for the model for HPAIV H5N6 surveillance data than that based on the HPAI H5N6 outbreak data. Significant risk factors and their impact differed slightly between the two models for HPAIV H5N6 surveillance data and reported HPAI H5N6 poultry outbreaks. In the model for HPAIV H5N6 surveillance positives, population density was identified as the most critical variable, which was followed by road density, poultry market density, mean diurnal range, and chicken density (Figure 2(a)). In the model for HPAI H5N6 poultry outbreaks, population density was also identified as the most vital variable for model construction, followed by road density, mean diurnal range, precipitation of the driest month, and chicken density (Figure 2(b)).

**Figure 3:**
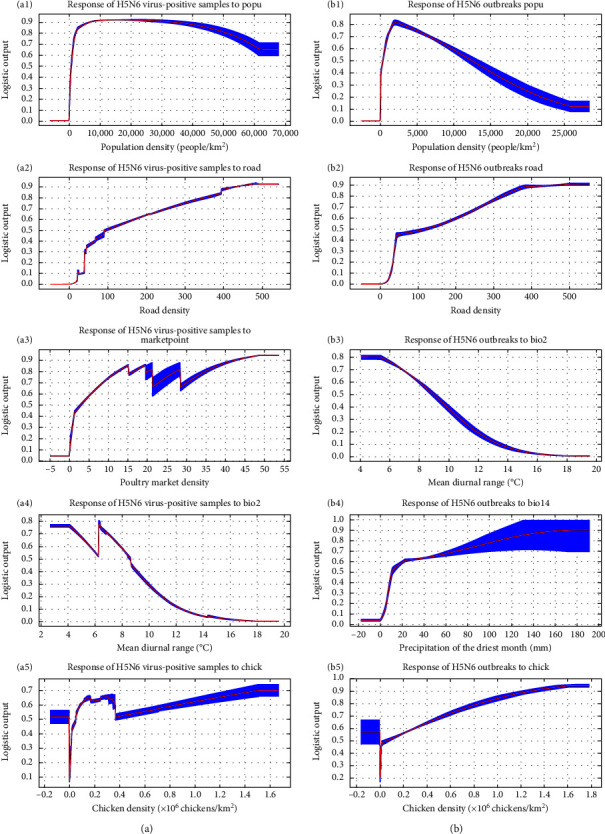
The response curves of key variables. The top five figures (a1–a5) are the response curves of key variables related to H5N6 virus-positive samples. The following five figures (b1–b5) are the response curves of key variables related to H5N6 outbreaks.

The response curves of key variables are shown in Figure 3. The horizontal axis of response curves represents the values of variables, and the vertical axis represents the predicted risk (range 0–1). A risk >0.5 is considered a high predicted risk. Based on the response curves of the model for HPAIV H5N6 surveillance data, the risk of HPAIV H5N6 presence rises rapidly with population density, peaking at a density of 17,661 people/km^2^ and remaining relatively constant until 20,326 people/km^2^. Subsequently, it gradually declines, remaining constant again at 61,767 people/km^2^. The risk of HPAIV H5N6 presence was positively associated with road density and poultry market density. In other words, the virus is more likely to be present in areas close to roads and poultry markets. Moreover, the HPAIV H5N6 presence risk appears to be constant when the mean diurnal range increases from 0 to 4.16°C, then gradually increases, and then plummets to a peak at 6.25°C. After that, the risk gradually decreases to 0. Unlike the previous variables, the risk of HPAIV H5N6 presence increases sharply with chicken density, peaks at 343,340 chickens/km^2^, and remains relatively stable until the density reaches 367,000 chickens/km^2^. After that, the risk abruptly drops and then slowly rises to a higher peak at 1,519,000 chickens/km^2^.

Based on the response curves of the model of the poultry outbreaks data, predicted outbreak risk increases significantly with population density, peaking at a density of 2,310 people/km^2^. Afterward, the outbreak risk gradually decreases to 0.5 as the population density increases to 12,446 people/km^2^ and then keeps decreasing. HPAI H5N6 poultry outbreaks were positively associated with road density, precipitation of the driest month, and chicken density and negatively with the mean diurnal range. The closer the area to the road, the higher the risk of H5N6 outbreaks. Furthermore, predicted outbreak risk increases with increasing precipitation in the driest month and remains constant after the precipitation of the driest month reaches 174 mm or more. Similarly, until chicken density reaches 162,681 chickens/km^2^, the outbreak risk of HPAI H5N6 outbreaks continues to increase and then remains constant for higher values. Contrarily, the outbreak risk appears to be constant for the mean diurnal range growing from 0 to 5.33°C, then decreases to a minimum risk at around 18.26°C.

**Figure 4:**
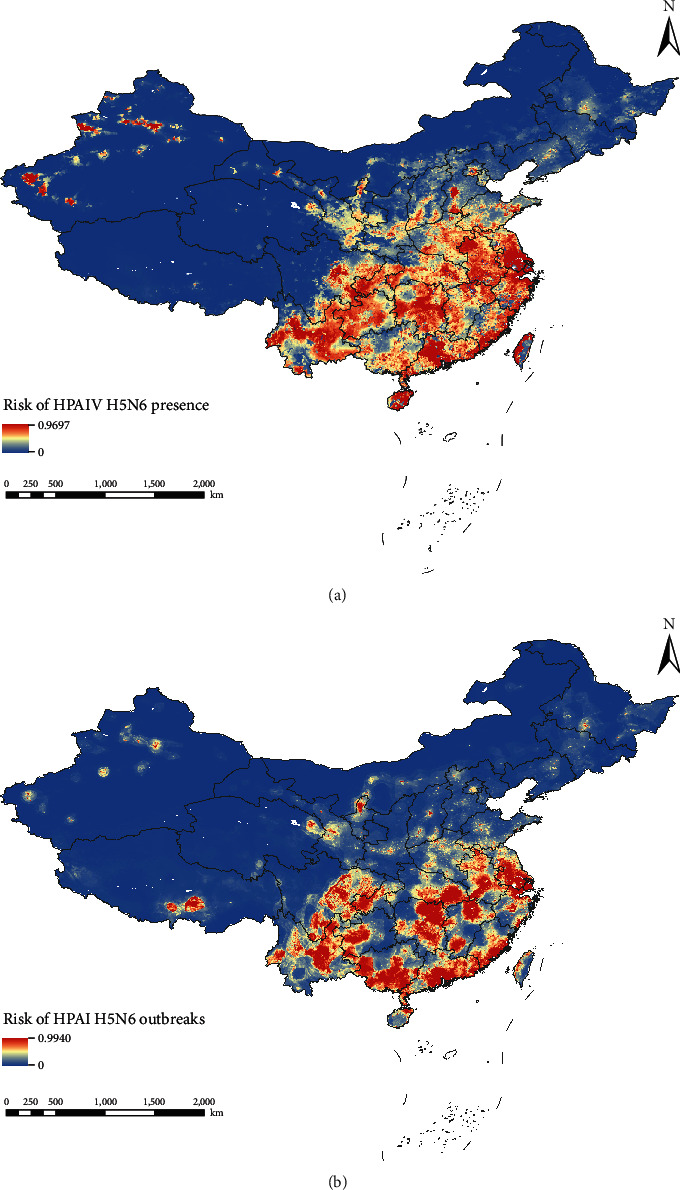
Risk maps of HPAIV H5N6 presence (a) and HPAI H5N6 outbreaks (b).

The predicted risk maps of HPAI H5N6 presence in China are shown in Figure 4. Overall, the high-risk areas of HPAIV H5N6 presence and HPAI H5N6 outbreaks are mainly located in Southern China. The risk areas for HPAIV H5N6 presence are focused in Eastern Yunnan, Western Guizhou, Eastern Sichuan, Western Chongqing, Southern Guangxi, most of Guangdong, Hong Kong, Macau, Southern Fujian, Eastern Hunan, a small area in Southern Jiangxi and a small area in the north, central and coastal parts of Zhejiang, Shanghai, Southern Jiangsu, Southern Anhui, Eastern Hubei, Northern Ningxia, Urumqi and Kashgar areas of Xinjiang, and Lhasa in Tibet. In contrast, except in Guangxi, Northern Ningxia, and Lhasa of Tibet, the map generated based on the outbreak data has a broader range of high-risk and medium-risk areas within the provinces mentioned above provinces than that based on surveillance data. Moreover, the model based on poultry outbreaks showed outbreak risk in Hebei, Shandong, Hainan, and Taiwan was higher than the risk of HPAIV H5N6 presence.

## 4. Discussion

In China, where approximately 67.8 billion poultry were produced in 2020, significant regional differences in ecology, livestock farming distribution, dietary habits of the population, and economic development have a consequential impact on the distribution, maintenance, and spread of infectious diseases, including HPAI H5N6.

Poultry farming in China is increasingly moving toward an intensive management model, which can reduce farming costs and bring more economic benefits [28]. Meanwhile, a nationwide vaccination campaign, mandatory for all poultry since 2005, protected from avian influenza subtypes H5 and H7. Since the first outbreak of HPAI H5N6 in China, a country with a vast and extensive poultry farming population, there have been only 46 poultry outbreaks in the past 9 years. Unlike the severe situation of HPAI H5N1 epidemic in China before, the effective control of HPAI H5N6 in China can be attributed to a combination of intensive farming and mass vaccination in poultry flocks. However, small-scale occurrences of outbreaks have existed with vaccine failure due to immune evasion and antigenic drift [29, 30], poor vaccination techniques and vaccine quality [31], and missed vaccinations (rare occurrences) [32]. In addition, immunization campaigns may have played an important role in driving the spread of AIV [33], with vaccinated animals potentially becoming silent virus carriers [34–36] and spreading AIV further through poultry markets and poultry trade [37]. Moreover, the national surveillance program for HPAIV of H5 subtype has repeatedly detected HPAIV H5N6 in poultry throat swabs, indicating that HPAIV H5N6 is still persistent in poultry flocks. The situation to prevent the spread of HPAIV H5N6 and outbreaks in poultry remains critical.

So far, spatial research aimed at determining the risk factors of highly pathogenic H5N6 virus has been conducted in South Korea [17]. However, the risk factors affecting the occurrence and transmission of HPAI H5N6 in China are still unclear. Based on the surveillance data and outbreak data, two MaxEnt models have been established separately to reveal for the first time the risk factors of HPAIV H5N6 existence and H5N6 outbreaks in China. Risk maps in China were also produced based on the model construction results.

Our study reported the distribution of HPAIV H5N6 presence and HPAI H5N6 outbreaks associated with areas of higher population density, closer proximity to roads, lower mean diurnal range, and higher chicken density. Humans played an important role in the transmission and outbreaks of HPAI H5N6. It can be explained by the higher likelihood of transmitting HPAIV H5N6 through the live birds trade and human activities exposed to live birds and the high probability of finding outbreaks in densely populated areas. At the same time, population density can be seen as a combination of other important risk factors. Areas with a high population tend to be located in the plains at low altitudes [38, 39]. These areas have a low mean diurnal range, a high demand for chicken products, a high poultry density, and a dense road distribution.

The reason for including road density in our study was that the influence played by roads in the H5N1 outbreaks in China [10] and Thailand [40] was well-established. However, the relationship between HPAI H5N6 and roads has yet to be discovered. Our study confirmed that road density played a positive role in HPAIV H5N6 presence and HPAI H5N6 outbreaks. It is clear that there are many limitations to using railways for poultry transport in China [41] and that waterways are not suitable for long-distance trade across China. Therefore, poultry transportation in China is often carried out by road, which is fast and can reach a wider area. When transporting live birds for a long distance, cross-infection of viruses is easily caused, expanding the range of infected groups with the high density of live birds in the carriage [41]. Some excreta or other HPAIV-contaminated tissue from live birds infected with HPAIV H5N6 can easily fall into the environment around roads during transport [42] and then be spread through human activities [43] or natural environmental conditions, causing infection and illness of susceptible bird flocks in the area around the road.

Two aspects were considered regarding the negative impact of the mean diurnal range on HPAIV H5N6 presence and HPAI H5N6 outbreaks. First, the low mean diurnal range at low altitudes [44] favors HPAIV H5N6 presence and HPAI H5N6 outbreaks. That was corroborated by the inclusion of altitude in our study, although altitude was not a significant factor associated with HPAI H5N6. The response curves for the altitude showed a gradual decrease in HPAIV H5N6 presence and HPAI H5N6 outbreaks with increasing altitude. Second, the transmission of HPAIV H5N6 and the HPAI H5N6 epidemic are more likely to occur in winter because the mean diurnal range in winter is smaller than in summer [45]. The 46 outbreaks of HPAI H5N6 collected in this study occurred most frequently in October and January, also proving our guess about the mean diurnal range.

Our study reported that chicken density has a certain impact on HPAIV H5N6 presence and HPAI H5N6 outbreaks. It can be seen from the jackknife test image of AUC that the chicken density is more important than the duck density for the HPAI H5N6 outbreaks. It completely explained that HPAIV H5N6 is more pathogenic to chickens than ducks. Interestingly, in the jackknife test image of AUC based on HPAIV H5N6 surveillance data, the chicken density is still more important than the duck density. It indicated that the transmission capacity of HPAIV H5N6 in chickens might be higher than in ducks, contrary to the conclusions of many previous studies [14, 46]. However, in a team's research on the avian influenza virus in the poultry market in China, it was found that one of the two major genotypes of HPAIV H5N6 in China is a gene box from the chicken, indicating that the ability of HPAIV H5N6 to spread among chickens has been enhanced [47]. It can support our discovery that chicken density is more associated with the presence and spread of HPAIV H5N6 than duck density. Additionally, chicken density can be regarded as a proxy variable of intensive breeding level. In recent years, the breeding mode of chickens in China has become more and more intensive and modern, while the breeding mode of ducks is mostly backyard and pond breeding [48]. In the background of intensive farming, the possibility of transmitting the virus to chickens through ducks and waterfowl is becoming increasingly low. On the contrary, the possibility of spreading the virus through human activities, live poultry trade, and long-distance transportation has increased.

Our study also reported different analysis results on risk factors of surveillance data and outbreak data. The HPAIV H5N6 presence was positively correlated with the poultry market density. Many previous studies have agreed that the poultry market is a beneficial place for the spread of HPAIV [15, 16]. In more detail, the Chinese people's habit of buying live birds [9], especially before the New Year, has dramatically increased the probability of cross-infection of viruses, enabling viruses to spread further through poultry trade and transport. Therefore, it is necessary to strengthen the virus surveillance and management of poultry markets. Besides, the HPAI H5N6 outbreaks positively correlated with the precipitation of the driest month. The abundant precipitation in the driest month can keep the water volume of wetlands and water bodies from drying up, creating an excellent resting environment for migratory birds [49]. Naturally, migratory birds infected with the virus can discharge the virus into the water with feces or secretions. Further, AIV can survive 4 days in water at 22°C and 30 days in water at 0°C [50, 51]. Domestic waterfowl in the same waterbody may not show clinical signs when they are exposed to AIV [52]. They will take the virus away from the resting place of migratory birds and spread it to other poultry [14], causing an epidemic outbreak.

The reason for separating HPAIV H5N6 infection data from HPAI H5N6 outbreak data is that there may be differences in the transmission mechanisms of HPAIV H5N6 and the occurrence mechanisms of HPAI H5N6. For example, some terrestrial birds that share the habitat of waterbirds and visit poultry farms for opportunistic foraging can become carriers of HPAIV H5N6 and transmit the virus undetected to other poultry populations without any clinical signs themselves [53]. Therefore, the spread of HPAIV H5N6 in mainland China may be invisible and difficult to monitor. In contrast, HPAI H5N6 outbreaks require several conditions, such as a suitable environment for the virus to survive and susceptible bird population in the area. HPAI H5N6 outbreaks are possible once the virus has been transmitted to this area. To summarize, the factors that influence the spread and occurrence of the disease may be distinct and need to be answered in this study.

This research has some limitations. The HPAI H5N6 outbreaks were obtained from an official source, which might suffer from underreporting of HPAI H5N6 outbreaks. If underreporting did exist in the data, many of the findings in our study could many of the findings in the study may not be the true epidemiology of the disease. Although pseudo-absence data were generated to reduce the sampling bias, there are other possible ways that bias in the outbreak and pseudo-absence data could also bias MaxEnt results.

It has been well-documented that the stopover sites of migratory birds during their migration are likely to be important vectors of AIV transmission between wild birds and poultry [54]. However, the objective data on the migratory routes of migratory birds are not available to us. Therefore, to demonstrate the influence of migratory birds in the occurrence and transmission of HPAI H5N6, this study incorporated waterbody—the stopover sites of migratory birds—into our model construction. In subsequent studies, our study will attempt to improve this aspect and explore more intuitively the impact of migratory bird migration on HPAI H5N6.

## 5. Conclusion

Based on HPAIV H5N6 surveillance data and HPAI H5N6 outbreak data, two MaxEnt models were established to determine the high-risk areas of HPAIV H5N6 presence and transmission and the high-risk areas of H5N6 outbreaks. HPAIV H5N6 presence was related to population density, road density, poultry market density, mean diurnal range, and chicken density. In contrast, HPAI H5N6 poultry outbreaks were associated with population density, road density, mean diurnal range, precipitation of the driest month, and chicken density. The high-risk areas of two models are mainly located in Southern China. This study can help animal epidemic prevention departments to carry out targeted prevention and control work in areas with the risk of HPAIV H5N6 transmission and HPAI H5N6 outbreaks.

## Figures and Tables

**Table 1:** Variables used in two models.

Variable description	Variable	Description	Included in model about HPAIV H5N6 presence	Included in model about reported HPAI H5N6 outbreaks
Bioclimate variables	bio1	Annual mean temperature		
	bio2	Mean diurnal range	Yes	Yes
	bio3	Isothermality		Yes
	bio4	Temperature seasonality	Yes	
	bio5	Maximum temperature of the warmest month	Yes	Yes
	bio6	Minimum temperature of the coldest month		
	bio7	Temperature annual range		
	bio8	Mean temperature of the wettest quarter	Yes	Yes
	bio9	Mean temperature of the driest quarter		
	bio10	Mean temperature of the warmest quarter		
	bio11	Mean temperature of the coldest quarter		
	bio12	Annual precipitation		
	bio13	Precipitation of the wettest month	Yes	Yes
	bio14	Precipitation of the driest month		Yes
	bio15	Precipitation seasonality	Yes	Yes
	bio16	Precipitation of the wettest quarter		
	bio17	Precipitation of the driest quarter	Yes	
	bio18	Precipitation of the warmest quarter		
	bio19	Precipitation of the coldest quarter		

Geographic variables	elev	Elevation	Yes	Yes
	Waterbody	Waterbody density	Yes	Yes

Social variables	Popu	Population density	Yes	Yes
	Chick	Chicken density	Yes	Yes
	Duck	Duck density	Yes	Yes
	Road	Road density	Yes	Yes
	Marketpoint	Poultry market density	Yes	Yes

## Data Availability

The datasets generated and/or analyzed during the current study are available from the corresponding author upon reasonable request.
